# Prognostic significance of bone marrow infiltration detected by PET-CT in newly diagnosed diffuse large B cell lymphoma

**DOI:** 10.18632/oncotarget.7616

**Published:** 2016-02-23

**Authors:** Jin-Hua Liang, Jin Sun, Li Wang, Lei Fan, Yao-Yu Chen, Xiao-Yan Qu, Tian-Nv Li, Jian-Yong Li, Wei Xu

**Affiliations:** ^1^ Department of Hematology, The First Affiliated Hospital of Nanjing Medical University, Jiangsu Province Hospital, Nanjing 210029, China; ^2^ Department of Nuclear Medicine, The First Affiliated Hospital of Nanjing Medical University, Jiangsu Province Hospital, Nanjing 210029, China; ^3^ Collaborative Innovation Center for Cancer Personalized Medicine, Nanjing Medical University, Nanjing 210029, China

**Keywords:** diffuse large B cell lymphoma, bone marrow involvement, PET-CT, survival

## Abstract

The aim of this study was to examine the prognostic value of bone marrow involvement (BMI) assessed by baseline PET-CT (PET(0)-BMI) in treatment-naïve patients with diffuse large B-cell lymphoma (DLBCL). All patients from a single centre diagnosed as DLBCL between 2005 and 2014 had data extracted from staging PET-CT (PET(0)-CT), bone marrow biopsy (BMB), and treatment records. The PET(3)-CT (PET-CT scan after cycle 3 of immunochemotherapy) was performed on all the patients with PET(0)-BMI positivity (PET(0)-BMI(+)). Of 169 patients, 20 (11.8%) had BMI on BMB, whereas 35 (20.7%) were PET(0)-BMI positive. Among PET(0)-BMI(+) patients, patients with maximum of standard uptake value (SUVmax) of bone marrow (SUVmax(BM)) more than 8.6 were significantly associated with high IPI score (3–5) (*P*=0.002), worse progression-free survival (PFS) and overall survival (OS) (*P*=0.025 and *P*=0.002, respectively). In the 68 stage IV cases, 3-year OS was higher in the patients with negative PET(0)-BMI (PET(0)-BMI(−)) than that with PET(0)-BMI(+) (84.2%±6.5% vs. 44.1%±8.6%; *P*=0.003), while 3-year PFS only shown a trend of statistic significance (*P*=0.077) between the two groups. Among the 69 patients of inter-risk of IPI (2–3), patients with PET(0)-BMI(+) had significantly inferior PFS and OS than that with PET(0)-BMI(−) (*P*=0.009 and *P*<0.001, respectively). The cut-off value of the decreased percentage of SUVmax(BM) between PET(0)-CT and PET(3)-CT (ΔSUVmax(BM)) was 70.0%, which can predict PFS (*P*=0.003) and OS (*P*=0.023). These data confirmed that along with the increased sensitivity and accuracy of identifying bone marrow by PET-CT, novel prognostic values of marrow involvement were found in patients with DLBCL.

## INTRODUCTION

Diffuse large B-cell lymphoma (DLBCL) is the most common subtype of non-Hodgkin lymphomas (NHL), which comprise approximately 5% of all malignancies in western countries [1]. The International Prognostic Index (IPI) is now widely used to predict prognosis for DLBCL patients [2]. Based on the recognized fact that bone marrow infiltration (BMI) categorized as an extranodal site, indicating the Ann Arbor stage IV, adversely affecting IPI scores, therapeutic strategy and clinical outcome [3], an accurate detection of BMI may play an important role in better managements of patients with DLBCL.

Positron emission tomography with computed tomography (PET-CT), a noninvasive whole-body metabolic imaging technique, can replace the traditional method of bone marrow biopsy (BMB) to detect bone marrow status in Hodgkin's lymphoma (HL) and DLBCL except DLBCL patient with a negative PET-CT for whom identification of occult discordant histology is clinically important [4–9]. Along with the increased sensitivity and accuracy of BMI detected by baseline PET-CT (PET(0)-BMI), more special attention should be paid to this special PET(0)-BMI positive (PET(0)-BMI(+)) subgroup. Actually, no data has been published until now.

The aim of this study was to examine the prognostic value of BMI assessed by baseline PET-CT (PET(0)-CT) in treatment-naïve DLBCL patients in four subgroups: (1) prognostic significance of BMI detected by PET(0)-CT in the whole cohort; (2) prognostic significance of BMI detected by PET(0)-CT in cases with stage IV disease; (3) prognostic significance of BMI detected by PET(0)-CT in cases with IPI score of 2–3; (4) prognostic significance of BMI detected by PET-CT after cycle 3 of immunochemotherapy (PET(3)-CT) in cases with PET(0)-BMI(+).

## RESULTS

### Patients' characteristics

The total number of the patients eligible for analysis was 169 (male-to-female ratio, 1). The study was approved by the Ethics Committee of the First Affiliated Hospital of Nanjing Medical University. Subjects provided informed consent according to institutional guidelines. The median age was 55 years (range 18–85 years) at diagnosis. Twenty-eight patients (16.6%) had an IPI score of 4–5, 77 (45.6%) had elevated lactate dehydrogenase (LDH), 108 (63.9%) were at stage III or IV according to Ann Arbor classification, 34 (20.1%) had an ECOG score of 2 or more, and 46 (27.2%) had more than one extranodal site of involvement (Table 1). As regard to first-line treatment strategies of all the 169 patients, 102 (60.3%) were treated with R-COHP-21 (rituximab plus cyclophosphamide, doxorubicin, vincristine and prednisone) with the median cycle of 6 (range 3–8) and 67 (39.7%) were treated with DA-EPOCH-R (dose-adjusted etoposide, doxorubicin, and cyclophosphamide with vincristine, prednisone, and rituximab) with the median cycle of 6 (range 3–8).

**Table 1:** Baseline characteristics according to BMI status by PET(0)-CT (N=169)

	N (%)	PET(0)-BMI(+), N=35 (N, %)	PET(0)-BMI(−), N=134 (N, %)	*P*
Male sex	94 (55.6)	24 (68.6)	70 (52.2)	0.090
Age >60 years	70 (41.4)	16 (45.7)	54 (40.3)	0.569
LDH >ULN	77 (45.6)	24 (68.6)	53 (39.6)	0.544
Extranodal site >1	46 (27.2)	18 (51.4)	28 (20.9)	<0.001
ECOG 2−4	34 (20.1)	15 (42.9)	19 (14.2)	<0.001
BMB(0)-BMI(+)	20 (11.8)	18 (51.4)	2 (1.5)	<0.001
**Stage**				<0.001
I	32 (18.9)	0 (0)	32 (23.9)	
II	29 (17.2)	0 (0)	29 (21.6)	
III	38 (22.5)	0 (0)	38 (28.4)	
IV	70 (41.4)	35 (100)	35 (26.1)	
**IPI**				<0.001
0–1	72 (42.6)	2 (5.7)	70 (52.2)	
2–3	69 (40.8)	21 (60.0)	48 (35.8)	
4–5	28 (16.6)	12 (34.3)	16 (11.9)	
Non-GCB	101 (59.8)	20 (57.1)	81 (60.4)	0.847

### BMI characteristics

Twenty patients (11.8%) had positive baseline BMI detected by BMB (BMB(0)-BMI), whereas 35 (20.7%) had PET(0)-BMI positivity. Thirty-three of the 35 PET(0)-BMI(+) patients showed a focal pattern and two a diffuse pattern, respectively. In PET(0)-BMI(+) patients, the meidan maximum of standard uptake value (SUVmax) was 11.2 (3.1–30.5); and the median SUVmax in cases with focal and diffuse involvement was 10.9 (3.1–30.5) and 16.6 (14.3–18.9), respectively. Baseline clinical characteristics of patients with or without BMI detected by PET(0)-CT were shown in Table 1. Significant differences (*P*<0.001) were observed between groups concerning the numbers of the extranodal sites and ECOG score, stage and IPI score.

Among the 33 PET(0)-BMI(+) patients with the focal patterns, 15 (45.4%) had bilateral iliac crests and other bone/bone marrow involvements distant from the bilateral iliac crests, 6 (18.2%) had unilateral iliac crests (four in the left and two in the right) and other involvements, 12 (36.4%) only had involvements in the other bone marrows distant from the bilateral iliac crests. The 15 cases with bilateral crest iliac involvements also had positive BMI detected by BMB (BMB(0)-BMI(+)); two with left unilateral crest iliac involvement, whose locations of BMB were also in the left, had the positive BMB(0)-BMI; four with right unilateral crest iliac involvement, whose locations of BMB were in the right, did not have positive BMB(0)-BMI and 12 without crest iliac involvement were all in the status of negative BMB(0)-BMI (BMB(0)-BMI(−)) (Table 2).

**Table 2:** The locations of BMI among the 35 patients with PET(0)-BMI(+)

Locations of PET(0)-BMI(+)		N	BMB-BMI(+)	Locations of the BMB
**Focal pattern**	Bilateral crest iliac and others	15	15	12 in left, 3 in right
	Right unilateral crest iliac and others	4	0	4 in left
	Left unilateral crest iliac and others	2	2	2 in left
	Others	12	0	8 in left, 4 in right
**Diffuse pattern**		2	1	2 in left

### Impact of PET(0)-CT findings on staging

According to the Ann Arbor classification, 32 (18.9%), 30 (17.8%), 39 (23.1%) and 68 (40.2%) patients were at stage I, II, III and IV, respectively, with PET(0)-CT. Taking both techniques of (PET(0)-BMI and BMB(0)-BMI) into account in this study, only two patients were upstaged by baseline BMB (One from II to IV, the other from III to IV) (Supplementary Table 1).

### Survival analysis

The follow-up results of all 169 patients enrolled in this study were at the end of Aug 2015 with the median follow-up of 38 months (12–113 months). Sixty-three patients (37.2%) progressed or relapsed and 36 (21.3%) died. In the whole crowd, the 3-year estimated progressive-free survival (PFS) and overall survival (OS) were 62.6%±3.9%, 81.0%±3.2%, respectively.

### Survival analysis according to PET(0)-CT findings in the whole cohort

**Figure 1:**
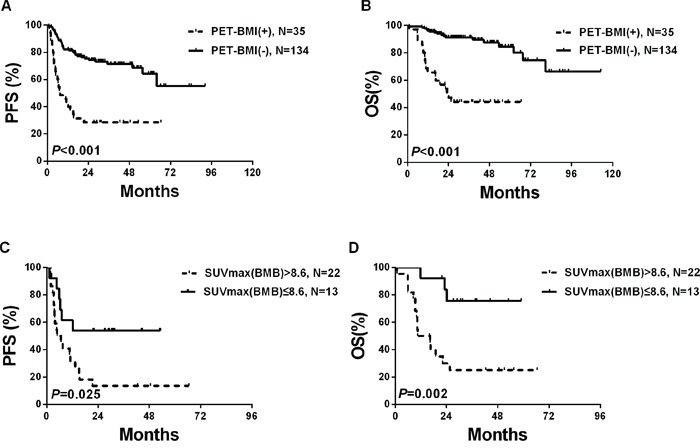
Survivals according to PET(0)-BMI status in the whole cohort

The PET(0)-BMI(+) patients had significantly inferior PFS (HR 3.96, 95% CI 2.38–6.59, *P*<0.001) and OS (HR 6.73, 95%CI 3.40–13.34, *P*<0.001) than the PET(0)-BMI negative (PET(0)-BMI(−)) patients (Figure 1A and 1B).

By univariate analysis, factors predictive of both OS and PFS included IPI score above 2, BMB(0)-BMI(+) and PET(0)-BMI(+). Regarding IPI factors, more than one extranodal site, elevated LDH level, Ann Arbor stage III or IV and ECOG score of 2 or more had predictive value (Table 3). Factors significantly associated with PFS and OS in univariate analysis were entered into multivariate analysis, and PET(0)-BMI(+) remained significant for OS (HR 2.90, 95% CI 1.21–6.96, *P*=0.017) while not for PFS (Table 3).

**Table 3:** Cox regression analysis for PFS and OS for all the patients with DLBCL (N=169)

Factors	PFS				OS			
	Univariate analysis		Multivariate analysis		Univariate analysis		Multivariate analysis	
	HR (95%CI)	*P*	HR (95%CI)	*P*	HR (95%CI)	*P*	HR (95%CI)	*P*
PET(0)-BMI(+)	3.96 (2.38–6.59)	<0.001	-	-	6.73 (3.40–13.34)	<0.001	2.90 (1.21–6.96)	0.017
BMB(0)-BMI(+)	4.49 (2.53–7.98)	<0.001	-	-	6.24 (3.06–12.73)	<0.001	-	-
IPI > 2	7.27 (4.19–12.63)	<0.001	3.12 (1.31-7.47)	0.010	9.02 (3.94–20.61)	<0.001	3.62 (1.01–13.03)	0.049
Age > 60 years	1.61 (0.98–2.64)	0.060			1.18 (0.61–2.27)	0.627		
Stage III or IV	6.08 (2.77–13.36)	<0.001	-	-	6.78 (2.08–22.12)	0.002	-	-
ECOG 2−4	2.79 (1.65–4.71)	<0.001	1.97 (1.12-3.47)	0.019	3.39 (1.75–6.55)	<0.001	-	-
LDH>ULN	4.68 (2.82–7.78)	<0.001	-	-	4.31 (1.96–9.48)	<0.001	-	-
Extranodal site > 1	3.15 (1.91–5.18)	<0.001	-	-	3.04 (1.58–5.86)	0.001	-	-

Of the 35 PET(0)-BMI(+) patients, ROC curve analysis shown the optimal cut-off value for SUVmax(BM) (SUVmax of bone marrow) was 8.6 (sensitivity 87.6%, specificity 68.3%, AUC 0.742, *P*=0.002). Using this cut-off threshold, the Kaplan-Meier survival curves for OS and PFS were made. Three-year PFS was higher for the patients with SUVmax(BM) ≤ 8.6 than the patients with SUVmax(BM) > 8.6 (53.8%±13.8% vs. 13.6%±7.3%; *P*=0.025). OS also differed significantly (*P*=0.002) between the two groups, with the estimates of 3-year OS at 75.5%±12.3% and 25.0%±9.5%, respectively (Figure 1C and 1D). Furthermore, patients with SUVmax(BM) > 8.6 were significantly associated with high IPI score (3–5) (*P*=0.002) and shown a trend to be related to elevated LDH levels (*P*=0.057) and advanced stage (III–IV) (*P*=0.089) (Supplementary Table 2).

### Survival of cases at stage IV (N=68) according to PET(0)-CT findings

Of the 68 cases at stage IV according to PET(0)-CT, 21 (30.9%) had BMI only, 33 (48.5%) had other extranodal diseases (OED) except bone marrow while 14 (20.6%) had both BMI and OED.

**Figure 2:**
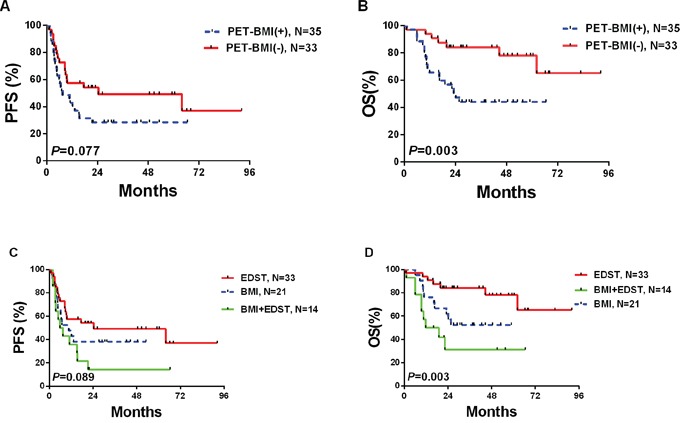
Survivals according to PET(0)-BMI status in cases at stage IV

In the 68 cases at stage IV, 3-year OS was higher in PET(0)-BMI(−) patients than PET(0)-BMI(+) patients (84.2%±6.5% and 44.1%±8.6%, respectively; *P*=0.003), while PFS only shown a trend of statistical significance (*P*=0.077) between the two groups, with estimates of 3-year PFS at 49.3%±9.2% and 28.6%±7.6%, respectively (Figure 2A and 2B); We further divided the stage IV cases into three categories: stage IV based on PET(0)-BMI(+) only (21 patients, 30.9%), stage IV based on PET(0)-BMI(+) and OED (14 patients, 20.6%), stage IV based on OED only (33 patients, 48.5%). The PET(0)-BMI(+) patients had similar OS and PFS to the cases with both PET(0)-BMI(+) and OED (Figure 2C and 2D).

### Survival of cases with IPI 2–3 disease (N=69) according to PET(0)-CT findings

Among the 35 patients with positive PET(0)-BMI, 2 (5.7%) were classified as low-risk (IPI, 1), 21 (60.0%) were inter-risk (2–3) and the other 12 (34.3%) were high-risk (4–5).

**Figure 3:**
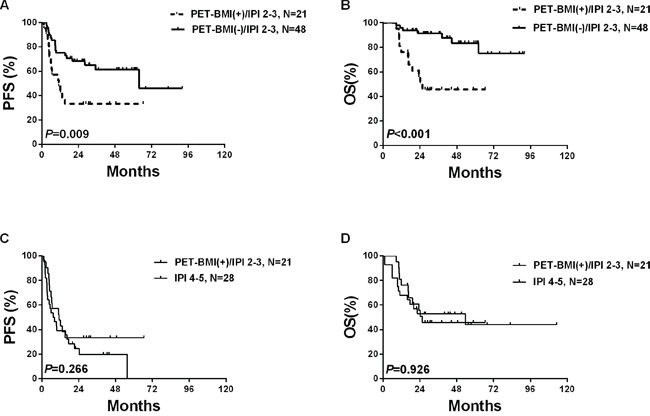
Survivals according to PET(0)-BMI status in cases with IPI score of 2–3

Among the 69 patients with IPI score of 2-3, the PET(0)-BMI(+) patients (N=21) had significantly inferior PFS and OS than the PET(0)-BMI(−) patients (N=48) (*P*=0.009 and *P*<0.001, respectively) (Figure 3A and 3B), and the 21 PET(0)-BMI(+) patients had similar PFS and OS to the patients with IPI scores of 4-5 (*P*=0.266 and *P*=0.926, respectively) (Figure 3C and 3D).

### Survival of cases with PET(0)-BMI(+) (N=35) according to PET(3)-CT findings

**Figure 4:**
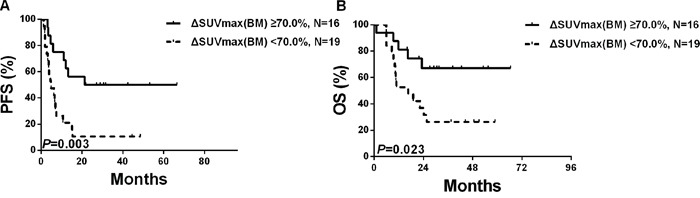
Survivals according to the PET(3)-CT based BMI status among the 35 patients with PET(0)-BMI(+)

Among the 35 PET(0)-BMI(+) patients, ROC curve analysis showed the optimal cut-off value for ΔSUVmax(BM) (the decreased percentage of SUVmax(BM) from PET(0)-CT to PET(3)-CT) was 70.0% (sensitivity 90.0%, specificity 78.2%, AUC 0.842, *P*=0.008). Using this cut-off threshold, the Kaplan-Meier survival curves for OS and PFS were made. Three-year PFS was higher in the patients (N=16) with ΔSUVmax(BM) > 70.0% than the patients (N=19) with ΔSUVmax(BM) ≤ 70.0% (50.0%±12.5% vs. 10.5%±7.0%; *P*=0.003) (Figure 4A). OS also significantly differed between the two groups, with the estimated 3-year OS at 67.0% ± 12.2% and 26.3%±10.1%, respectively (*P*=0.023) (Figure 4B).

## DISCUSSION

Based on the published data [10–12], the new Lugano classification recommended that PET(0)-CT staging in DLBCL can accurately detect BMI and that BMB was only for those with a negative PET-BMI for whom occult discordant histology was clinically important [4, 9, 13, 14]. However, along with the increased sensitivity and accuracy of BMI detected by PET(0)-CT, the clinical significance of BMI is more than just adversely affecting stage and, consequently, the IPI. To the best of our knowledge, this is the first comprehensive analysis to date investigating the new prognostic values of PET(0)-CT assessed BMI in the different subgroups.

In our study of 169 unselected patients treated with R-chemotherapy, PET(0)-BMI(+) occurred with an overall prevalence of 20.7%, in keeping with previous studies (range, 11% to 27%) [1, 9–12, 15–18]. Berthet et al. [10] performed a study on 142 DLBCL patients and observed that there were only 6 BMB positive patients in 31 patients with focal bone marrow uptake. Cortes-Romera et al. [11] reported 74 DLBCL patients in whom BMB missed 8 of 19 patients with focal bone marrow uptake. In the present study, 16 of 18 BMB(0)-BMI(+) patients had focal skeletal PET(0)-CT lesions while 19 of 35 PET(0)-BMI(+) patients had negative BMB(0)-BMI. In our study, 15 patients had bilateral crest iliac involvement, and all were BMB positive; Among the 6 patients with unilateral crest iliac involvement, the concordant results between BMB(0)-BMI and PET(0)-BMI would be observed only in patients with the consistent lesions of BMB and focal crest iliac uptake lesion. Patients with focal skeletal uptake distant from the bilateral crest iliac were all BMB(0)-BMI negative. Thus, the literature and our results consistently suggested that BMB might miss focal bone marrow involvements in patients with discordant locations between BMB and unilateral crest uptake, and in patients with skeletal uptake distant from the bilateral crest iliac.

In our study, PET(0)-CT only missed few patients (2 patients) with positive BMB(0)-BMI and negative PET(0)-BMI due to the discordant histology, which was in line with the reported prevalence [1, 10–12, 15]. One patient had already been staged as III according the PET(0)-CT imaging. Only one patient was upstaged from II to IV by positive BMB(0)-BMI. This patient was reevaluated from IPI score of 1 to 2. That's to say, we must do 169 bone marrow biopsies to catch two BMIs missed by PET(0)-CT while we can catch another 19 BMIs missed in BMB by performing 169 PET(0)-CT scans (*P*=0.001).

The presence of concordant BMI by BMB has been associated with a poor outcome in DLBCL patients in a number of prior studies [17, 19–22]. The impact of BMI assessed by PET(0)-CT has also been related to a poor outcome [9, 10]. Berthet et al. [10] showed that BMI assessed by PET(0)-CT was an inferior predictor of PFS independent of IPI, which was similar in our study. In our study, the median SUVmax(BM) of the PET(0)-BMI(+) patients was 11.2 (3.1–30.5), which was higher than 7.8 (2.2–29.3) in high grade B-cell NHL (232 patients: 155 was DLBCL) reported by Chen-Liang et al. [15]. ROC curve analysis showed that 8.6 was the optimal cut-off value for SUVmax(BM) to discriminate the PET(0)-BMI(+) patients into two risk groups. Patients with SUVmax(BM) less than 8.6 had similar PFS and OS to the patients with negative PET(0)-BMI (data not shown). This was mainly due to patients with SUVmax(BM) more than 8.6 were associated with unfavorable clinical parameters shch as advanced stage, elevated LDH level and high IPI score. Furthermore, we found that computation of ΔSUVmax(BM) leads to better outcome prediction for patients with PET(0)-BMI(+) and better reproducibility among the observers. Thus, this was the first time to date to evaluate the optimal cut-off value for SUVmax(BM) and ΔSUVmax(BM) to risk stratification for patients with positive PET(0)-BMI. Confirmation of our observations in a larger cohort is needed.

Khan et al. [12] identified that cases with a positive BMI by PET(0)-CT had outcome comparable to other patients with stage IV disease without positive BMI based on data of 130 DLBCL patients. In contrast, results from our center showed that a statistic significance for OS and trend significance for PFS were observed for PET(0)-BMI(+) patients compared PET(0)-BMI(−) patients in 68 cases at stage IV. We further divided the reasons for stage IV into three categories. Nearly 50% of the patients were at stage IV based on OED only, and no significance was observed between patients with “PET(0)-BMI(+) only” and “PET(0)-BMI(+) together with OED”. It gives us the clinical implication for the first time to date that only if patients with PET(0)-BMI(+), will have a worse outcome.

On the basis of previous reports, quite a large number of PET(0)-BMI(+) patients were IPI score 2–3 [10, 12], along with the increased sensitivity of BMI detected by PET(0)-CT. In our study, 21 (60.0%) PET(0)-BMI(+) patients were IPI 2–3, and among patients at progression, relapse, or death, more than 50% were IPI 2–3. Further risk-stratification was needed for intermediate risk-group. Based our results, we found that PET(0)-BMI can distinguish the patients with relatively poor survivals from the whole group of IPI 2–3. The patients with PET(0)-BMI(+) and IPI 2–3 had similar survival to the high risk patients. Thus, patients with PET(0)-BMI(+) in the intermediate risk-group should be managed as high-risk patients [5]. Risk-stratification capacity of IPI can be enhanced by combined with PET(0)-BMI. If validated, this is of great importance in clinical practice.

To summarize, this retrospective study focused on the comprehensive and further analysis of the predictive significance of bone marrow involvement in different DLBCL subgroups. Our data raise several important issues: (1) The bone marrow status assessed by baseline PET-CT is an independent predictor of OS; (2) The optimal cutoff value of SUVmax(BM) and ΔSUVmax(BM) to predict the outcomes for baseline PET-BMI(+) patients was 8.6 and 70.0%, respectively; (3) In patients at stage IV, worse survival outcomes were observed in patients with BMI than that without BMI; (4) Patients with baseline PET-BMI(+) in the intermediate risk-group of IPI should be managed as high-risk patients.

## MATERIALS AND METHODS

### Patients

Patients who were diagnosed with DLBCL between April 2005 and July 2014 were included in the study if both PET(0)-CT and BMB were performed and no malignancy other than DLBCL was present at the time of examination. The PET(3)-CT was performed on all PET(0)-BMI(+) patients. The patients below were excluded: patients had received chemotherapy before the PET(0)-CT scan or hematopoietic growth factor before any PET-CT scan; and patients had the interval more than 30 days between baseline BMB and PET(0)-CT. The clinical characteristics were obtained by review of medical records of the patients.

### BMB

All of the bone marrow samples were obtained from unilateral iliac crest. The presence of BMI was confirmed by comparing the morphology of bone marrow to extra marrow tissues including lymphoid nodule, spleen and others. The monoclonal antibodies of CD3, CD20, PAX5, CD79a, CD10, Bcl-6, Bcl-2, Mum-1 and Ki- 67, which were used to identify the abnormal large and small B-cells, were performed on the standard immunohistochemical staining. The definition of the positive BMB(0)-BMI was either the large or small B-cells evaluated by our experienced hemapathologists.

### PET-CT image acquisition

PET-CT examination was performed with an integrated scanner (Siemens biograph 16; Siemens). ^18^F-fluorodeoxyglucose (^18^F-FDG) was produced by CTI RDS I I I cyclotron (GE; USA) and the radiochemical purity was more than 95%. Each patient had to fast for at least 6 h and the blood glucose level must be less than 200 mg/dL before intravenous injection of ^18^F-FDG at the dose of 3.7-5.5 MBq/kg body weight and been suggested to drink about 1,000 mL water after injection. PET-CT scan was begun about 60 min after injection and the range was from the skull base to the middle of the femur. CT acquisition parameters were as follows: 120 kV and 200 reference mAs; dynamic dose control mode (Caredose 4D); 1.5 mm detector collimation and 5.0 mm slice thickness. PET parameters: 3D emission scan, 2 min per bed position; 6-7 beds, ordered-subset expectation maximization (OSEM) reconstruction. CT scan data was used for attenuation correction of PET image.

All scans were re-examined by an experienced radiologist who was unaware of both clinical and BMB findings. In addition, scans of patients with the inconsistent results at diagnosis (PET(0)-BMI(−) and BMB(0)-BMI(+), BMB(0)-BMI(−) and PET(0)-BMI(+)) were reviewed by another radiologist who was blind to the original PET(0)-CT scan and histopathology results.

The final diagnosis of BMI assessed by PET(0)-CT [10]: (i) Focal patterns of bone marrow uptake showing an increased activity higher than in the liver; (ii) Diffuse patterns of bone marrow uptake confirmed by guided biopsy or targeted MR imaging or concomitant disappearance of bone marrow uptake and uptake in other lymphoma lesions on PET(3)-CT after the standard immunochemotherapy.

### Data analysis

We report median and range values for continuous variables and percentages for categorical variables. OS was defined as interval from diagnosis to death owing to any cause by the end of follow-up (Aug, 2015). PFS was defined as interval from diagnosis to first progression, relapse.

Kaplan-Meier survival curves with the Log-rank test were performed to analyze PFS and OS, according to the absence or presence of BMI according to PET(0)-CT in different subgroups. Univariate and multivariate Cox proportional hazards regression models were performed for IPI score and factors, the status of BMB(0)-BMI and PET(0)-BMI. Receiver-Operator Characteristic (ROC) curve analysis and the area under the ROC curve was used to evaluate the optimal cut-off value of SUVmax(BM) and ΔSUVmax(BM) for patients with PET(0)-BMI(+). Statistical analysis was performed using MedCalc for Windows, version 12.0.4.0 (MedCalc Software, Mariakerke, Belgium). A P-value of <0.05 was considered to be significant.

## SUPPLEMENTARY TABLES


