# Supplementary material for: Prognostic significance of bone marrow infiltration detected by PET-CT in newly diagnosed diffuse large B cell lymphoma
Source: Oncotarget. 2016 Feb 23;7(14):19072–80. doi: 10.18632/oncotarget.7616 (PMC4951353; doi:10.18632/oncotarget.7616)
Supplement: Supplementary file 1 [file oncotarget-07-19072-s001.pdf]

SUPPLEMENTARY TABLES

Supplementary Table S1: Comparison of staging according to PET-CT or the combination of PET-CT + BMB in 169 patients with DLBCL

	Stage				
	PET-CT+BMB				
PET-CT	I	II	III	IV	Total
I	32	0	0	0	32
II	0	29	0	**1**	30
III	0	0	38	**1**	39
IV	0	0	0	68	68
Total	32	29	38	70	169

Supplementary Table S2: Baseline characteristics according to SUVmax(BM) status by PET(0)-CT (N=35)

	SUVmax(BM) > 8.6, N=22 (N, %)	SUVmax(BM) ≤ 8.6, N=13 (N, %)	*P*
Male sex	14 (64)	10 (77)	0.478
Age >60	11 (50)	5 (38)	0.727
LDH >ULN	18 (82)	6 (46)	0.057
Extranodal site >1	13 (59)	5 (38)	0.305
ECOG 2−4	12 (55)	3 (23)	0.089
Stage III-IV	22 (100)	22 (100)	1.000
IPI 3−5	21 (95)	6 (46)	0.002
BMB(0)-BMI(+)	15 (68)	3 (23)	0.015
